# The functional connectivity of basal forebrain is associated with superior memory performance in older adults: a case-control study

**DOI:** 10.1186/s12877-022-03226-w

**Published:** 2022-06-24

**Authors:** Shu-hong Jia, Zhi Zhou, Wen Shao, Xiao Zhou, Shuang Lv, Wen Hong, Dan-tao Peng

**Affiliations:** 1 Department of Neurology, China-Japan Friendship Hospital, Beijing, China; 2 Department of Radiology, China-Japan Friendship Hospital, Beijing, China

**Keywords:** Superagers, Memory, Basal forebrain, Resting-state functional MRI, Functional connectivity

## Abstract

**Background:**

Aging is related with memory deterioration. However, some older adults demonstrate superior performance compared to age- and education-matched adults, who are referred to as superagers. To explore the neural mechanisms that mediate their unusually successful memory is important not only for the ameliorate the effects of aging in brain, but also for the prevention of neurodegenerative diseases, including Alzheimer’s disease. This case-control study is aimed to investigate the effects of volume and function of basal forebrain cholinergic neurons on the cognition of superagers.

**Methods:**

The morphometric and resting-state functional MRI analysis, including 34 superagers and 48 typical older adults, were conducted. We compared the basal forebrain gray matter density and related resting-state functional connectivity (FC) in the two groups. To investigate the relationship of FC with cognition, we measure the correlation of significant altered FC and individual cognitive domain.

**Results:**

No significant differences of gray matter density was observed between superagers and typical older adults. The superagers had stronger cortical FC of Ch1-3 with left putamen and insular cortex. The strength of FC positively correlated with global cognition, memory and executive function.

**Conclusions:**

These findings demonstrated that the stronger FC of basal forebrain correlated with specific cognitive difference in global cognition and domains of memory and executive function in superagers.

## Background

Aging is related with decline of cognitive function, particularly memory deterioration. However, some older adults are able to demonstrate superior memory performance compared to age- and education-matched adults [1, 2], or demonstrate comparable performance to middle-aged or even younger adults [3, 4]. They were referred to as superagers [1, 5–9] or successful agers [10–12].

As aging is associated with gradual loss of brain micro- and macro- structure [13], the maintenance of superior memory suggests the resistance against conventional age-associated neurodegeneration and consequent cognitive decline. Previous studies found that individuals with superior memory had greater thickness of cortical regions which mainly located within the default mode and salience networks [7, 9], and other study found that superagers had thicker cortex in anterior cingulate cortex [14]. A longitudinal study also showed that the superagers had lower rate of cortex atrophy compared to typical older adults group [8].

In the society of global aging, understanding the neural mechanism of superior performance is important for the prevention of neurodegenerative diseases, including Alzheimer’s disease (AD). Extensive research has shown that the cognitive impairment, including memory decline, is a multisystem etiology in AD. Previous pathological studies found prominent cholinergic cell loss in the nucleus basalis of Meynert (NbM), supporting the “cholinergic hypothesis” to explain the impairment memory in AD [15]. Other research also confirmed the cholinergic neurotransmission contribute to the cognitive function, especially in attention and memory [16]. Cholinergic neurons which projected to the cortex mainly located in the basal forebrain. The basal forebrain is divided into different nuclei with distinct connections, that project to the hippocampus (medial septal nucleus and nucleus of the vertical limb of the diagonal band, Ch1-2), the olfactory bulb (nucleus of the horizontal limb of the diagonal band, Ch3), and the cortex and amygdala (the nucleus basalis of Meynert (NbM), Ch4, [17]. Cholinergic activity associated with basal forebrain is crucial for several cognitive processes, including memory [18–20].

Though it is well established that neurodegeneration especially affects cholinergic neurons in AD, up to now, the degree of integrity of cholinergic systems contribute to the memory performance in normal aging adults is still unclear. Development of neuroimaging provides a tool to quantify the volume and cholinergic denervation of basal forebrain in vivo. A recent study using MRI found that cholinergic forebrain structure is vulnerable to negative effects of aging. Even in healthy elderly people with stable cognition, the annual atrophy rate of the basal forebrain is approximately three times the overall gray matter atrophy rate [21]. Using PET with molecular tracer of acetylcholinesterase activity, mesiotemporal cholinergic deficit was found correlating with verbal memory in healthy elderly adults [22].

In this study, we hypothesized that differences in morphology and functional connectivity of basal forebrain cholinergic system between superagers and typical older adults may contribute to the inter-individual variability in cognitive performance. First, we used structural MRI to quantify structural changes in the different nuclei of the basal forebrain. Second, we used resting state functional MRI (rsfMRI) to quantify the cerebral functional connectivity (FC) of Ch1-3 and Ch 4, respectively. Third, we hypothesized that structural or functional changes were clinically relevant as they correlated with cognitive decline, the relationships between cognitive tests scores and the measure of structural and FC of each nuclei were evaluated.

## Methods

### Study design and participants

This was a retrospective case-control study. Participants were recruited from the family members of patients in memory clinic of China-Japan Friendship Hospital from 2014 to 2020. Participants who underwent structural and rs-fMRI images were recruited. In this study, the Mini-Mental State Examination (MMSE) was used as a general cognitive screening measure, participants with MMSE score lower than 24 were excluded. The sample was further restricted with these exclusion criteria: 1) age<60 years (the selection of age cut-off is described in detail in “Discussion”); 2) history of significant neurological disorder, including stroke, head trauma, intracranial mass, and epilepsy; 3) history of psychiatric disorders, including depression and schizophrenia; 4) left handed.

### Neuropsychological assessment and classification

The definition of superagers is defined according to the memory performance with the delayed recall score (30 min) of Rey Auditory-Verbal Learning Test (RAVLT). The superagers were required to perform higher than 1 standard deviation (SD) of the mean score for their age, gender and education [23]. In addition, superagers were required to perform within 1 SD of the average range for their age and education group on other non-memory cognitive subdomains, including the Trail Making Test Part A (TMT-A) for attention [24], the 30-item Boston Naming Test (BNT )[25] for language, TMT-B for executive function and Wechsler Adult Intelligence Scale (WAIS)-III Block Design for visuospatial function.

Age, gender and education level matched typical older adults met the following criteria: their test scores falling within 1 SD of the average range for their age and education according to published normative values.

### MRI data acquisition

The three-dimensional T1-weighted MRI and rs-fMRI images were acquired using a 3.0 T MR imaging system (GE Healthcare, Discovery MR750, Milwaukee, WI, USA) at the Department of Radiology, China-Japan Friendship Hospital. The parameters of T1 images with fast spoiled gradient-echo sequences (FSPGR) were as follows: repetition time (TR) = 6.9 ms, echo time (TE) = 3.0 ms, FOV = 256 mm × 256 mm, acquisition matrix =256 × 256, slice thickness = 1.0 mm, and flip angle = 12°. The parameters of axial rs-fMRI data were as follows: TR = 2000 ms, TE = 30 ms, field of view (FOV) = 240 mm × 240 mm, in plane matrix = 64 × 64, slice thickness = 3.0 mm, 33 slices, flip angle = 90°, and 240 volumes.

### Imaging preprocessing

We use the Data Processing Assistant for Resting-State fMRI (DPARSF) to preprocess the 3D T1-weighted MRI and rs-fMRI images [26]. The first 10 volumes were dropped for signal equilibrium. The remaining 230 volumes underwent correction for time slicing and realigned to the first image. To evaluate head movement, we used the 6 rotations/translations parameters and calculate the frame-wise displacement (FD) (Jenkinson) [27]. The FD of all the participants was 0.30 ± 0.12 mm. The images of 2 participants (1 superager and 1 typical older adult) were excluded because of the excessive head motion (including greater than 2.0 mm, greater than 2.0° angular rotation, or mean FD > mean FD +2SD). There was no significant difference in FD between the two groups (superagers: 0.28 ± 0.10 mm, typical older adults: 0.28 ± 0.12 mm; T-value estimated by two sample t-test was 0.36, *p* = 0.72) after removing the 2 subjects. The structural T1-weighted images were registered to the corresponding functional images and were then segmented into gray matter (GM), white matter (WM), and cerebrospinal fluid tissue (CSF) probabilistic maps for spatial normalization. The GM template for structural T1 image was derived from the whole image data set with DARTEL (Diffeomorphic Anatomical Registration using Exponentiaed Lie algebra) technique [28]. Nonlinear warping of the segmented images was performed to match the Montreal Neurological Institute (MNI) space DARTEL template. Then, the EPI images were smoothed with a 6-mm FWHM (full width at half maximum) Gaussian filter. To remove the high-frequency noise and low-frequency drifts, linear detrending and temporal band-pass filtering (0.01–0.1 Hz) were applied. Finally, the WM and CSF signal, the linear term, and the 6 head motion parameters and their derivatives were regressed out as nuisance variables.

### Seed region-of-interest

**Fig. 1:**
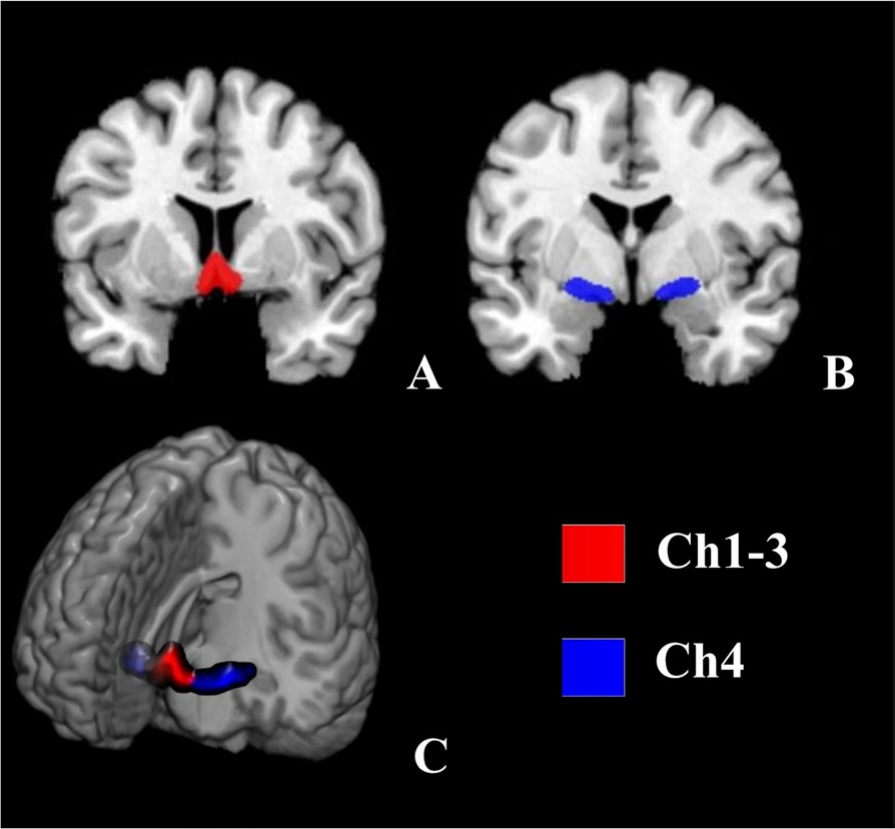
The seeds of the basal forebrain. The image was overlapped by the seeds. The different colors show the different parcellation

The comprehensive basal forebrain region-of-interest (ROI) was defined from probabilistic maps in the SPM Anatomy Toolbox [29]. The probability map was warped to fit the template created during DARTEL. Ch1, Ch2 and Ch3 (Ch1-3) were segmented together because they were difficult to delineate one from another and they all project to the limbic system (Fig. 1).

Normalized masks of Ch1-3 and Ch4 were taken as seed ROIs to test our hypotheses. The GM density of each part of basal forebrain was obtained from the atlas separately.

These masks were then resampled to a 3 mm isotropic to match the voxel size of the rs-fMRI images. For each subject, the voxel of each seed was extracted to obtain the average seed point time series. A correlation coefficient map of each seed was created by correlating the coefficients between the reference time series and the time series from all other cerebral voxels, which was then transformed to Fisher *z*-values.

### Statistical analysis

SPSS 23.0 (IBM Corp., Chicago, IL, USA) was used for data analysis. The normality of distribution for demographic and clinical variables were checked using Kolmogorov–Smirnov tests. Variables revealing normal distribution and gender were analyzed with Student’s t test and Chi-square test, respectively, at a significant level of 0.05.

The preprocessed T1 images were subsequently entered into a voxel-based morphometry (VBM) analysis integrated in DPARSF and implemented in Matlab R2014a (MathWorks, Natick, MA, USA). The unpaired two sample t-test analysis was performed to explore the GM density differences between two groups of the whole brain. Furthermore, for the volume of Ch1-3 and Ch4, the GM density of Ch1-3 and Ch 4 were compared with between-group differences. Age, gender, and years of education were included as nuisance covariates. As the voxel-wise GM density maps were modulated in normalization; the intracranial volume was not used as the covariate.

First, to explore the whole brain FC of the nucleus of Ch1-3 and Ch4, one sample t-test of the z-value maps were performed in all participants. The clusters surpassing a threshold of 0.001 (uncorrected for multiple comparisons) were used as a mask in the analysis of the next unpaired two sample t-test analysis. Second, to investigate the difference FC between two groups, a voxel-wise two sample t-test were conducted with the z-value maps between superagers and typical older adults within the mask which is produced in the previous step. Age, gender, education level, head movement parameters, and GM density of each ROI were used as covariates. False discovery rate (FDR) correction was performed with a threshold of 0.05. The significant clusters were overlaid onto the template during DARTEL procedure.

The z-values of FC were extracted from the significant clusters. We also check the correlation of significant different FC with global cognition and each individual cognitive subdomain (MMSE for global cognition, Delay recall of RAVLT for memory, TMT-A for attention, BNT for language, TMT-B for executive function and WAIS-III Block Design for visuospatial function) using the Pearson’s correlation, with age, gender, and education level as nuisance covariates. *p* <  0.05 was considered statistically significant. Furthermore, due to the small sample size of each group, for an exploratory analysis, the group with superagers and group with typical older adults were combined together to investigate the correlation.

## Results

### Demographic and neuropsychological results

**Fig. 2:**
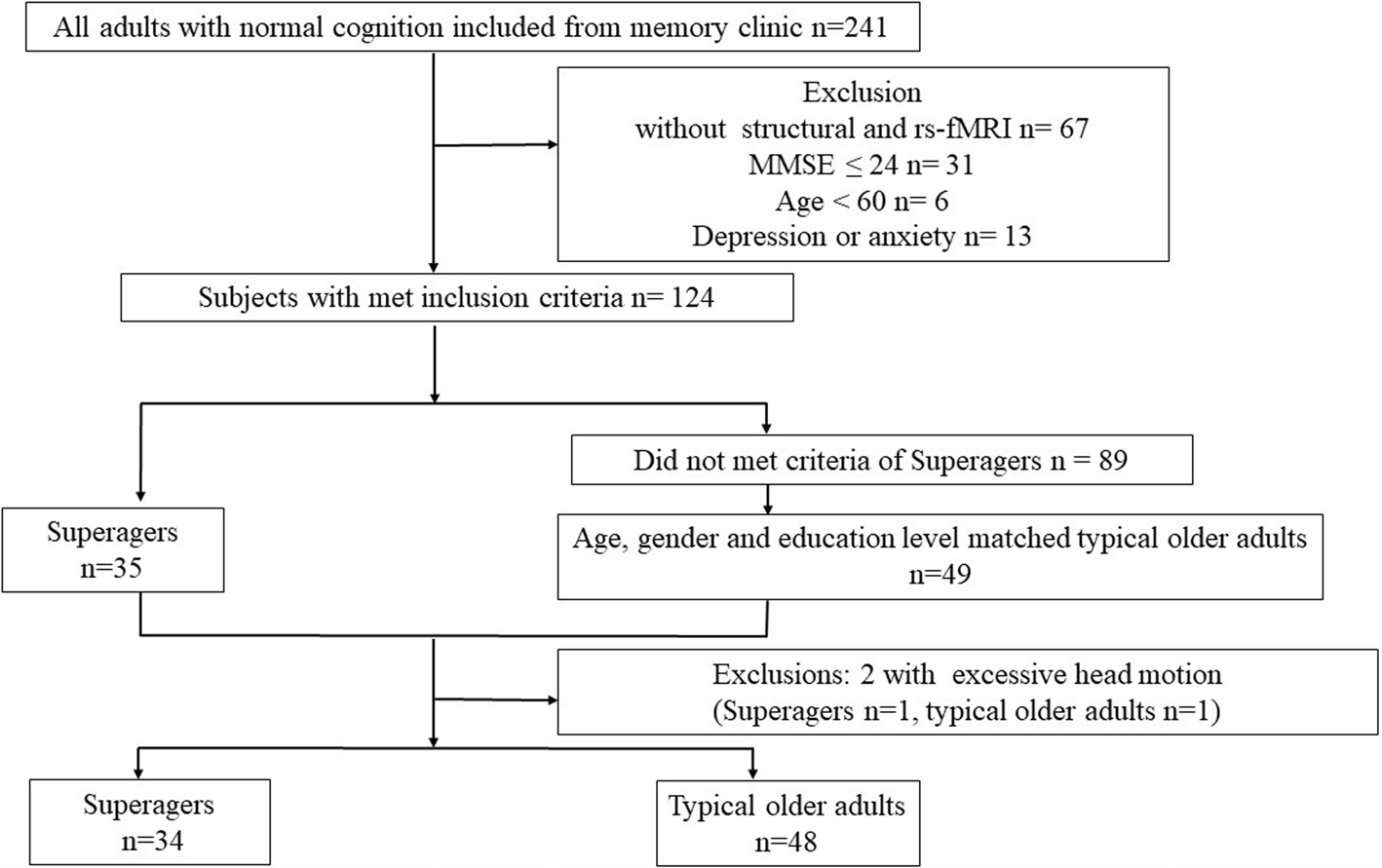
Diagram showing the number and flow of subjects in this study. Abbreviations: rs-fMRI: resting-state functional MRI; MMSE: Mini-mental State Examination

From June 2014 to December 2020, 34 superagers and 48 Typical older adults were recruited with the aforementioned procedures (Fig. 2). Table 1 shows the demographic and neuropsychological data. The group with superagers had significantly higher scores than group with typical older adults on general cognitive test and executive function.

**Table 1:** Demographic and neuropsychological data

	Superagers (***n*** = 34)	Typical older adults (***n*** = 48)	***t***	***p***
Age (y)	68.47 (6.51)	70.58 (5.82)	−1.54	0.13
Education in year (SD)	13.84 (1.94)	13.35 (3.10)	0.81	0.42
Gender (% male)	12 (25%)	12 (35.3%)	–	0.313
MMSE Raw (total = 30)	29.03 (1.03)	28.35 (1.41)	2.38	**0.02**
RAVLT Delay-Recall Raw (total = 15)	11.15 (0.79)	7.81 (1.51)	11.78	**0.00**
BNT-30 Raw	28.12 (1.81)	27.49 (2.48)	1.26	0.21
Trail-Making Test Part A Raw (sec)	52.33 (16.71)	53.81 (19.98)	−0.35	0.73
Trail-Making Test Part B Raw (sec)	102.42 (54.62)	138.49 (84.77)	−2.13	**0.04**
WAIS-III Block Design	42.44 (9.33)	38.04 (10.43)	1.99	0.05

*Abbreviations*: *MMSE* Mini-Mental State Examination, *RAVLT* Rey Auditory-Verbal Learning Test, *BNT* Boston Naming Test, *WAIS* Wechsler Adult Intelligence Scale. Bold means *p <* 0.05

### Whole brain and basal forebrain morphometry

The whole brain VBM did not show any significant difference between two groups. And no significant difference in the GM density of any nuclei of basal forebrain was found between groups with predefined threshold.

### Whole brain FC of the nucleus of Ch1-3 and Ch4

**Fig. 3:**
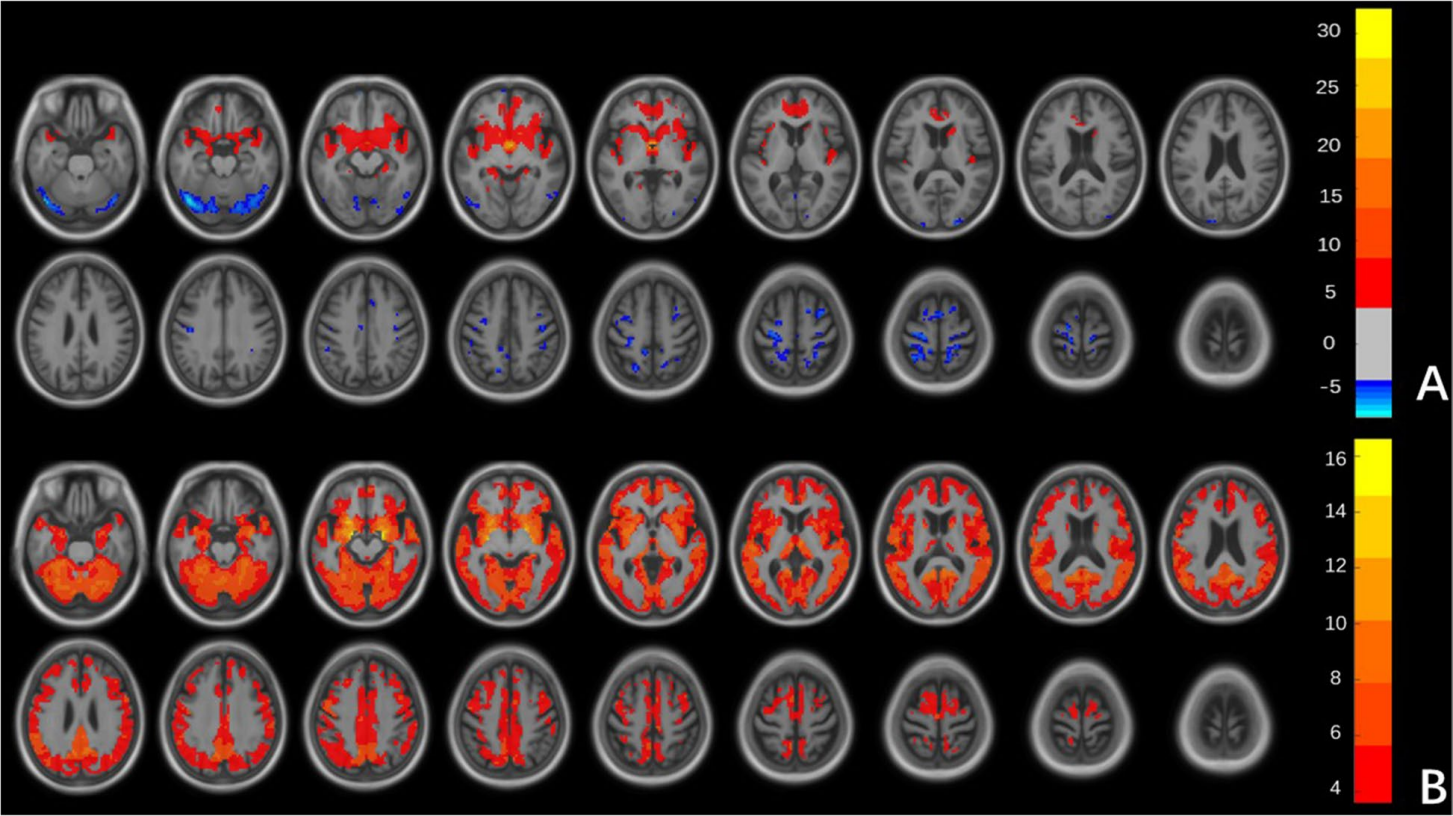
Statistical parametric map of the one-sample test in all participants. Brain areas that show positive (warm color) and negative (cool color) functional connectivity to the basal nucleus of Ch1-3 (**A**) and Ch 4 (**B**), respectively

For each ROI, the results of one sample t-test of the Z maps across the group are presented in Fig. 3. The Ch1-3 showed positive FC with bilateral medial prefrontal cortex (MPFC), medial orbitofrontal cortex (MOFC), inferior temporal cortex, insula, hippocampus and parahippocampal gyri, thalamus, and basal ganglia. The Ch1-3 showed negative FC with dorsolateral prefrontal cortex, frontal eye field, and posterior parietal cortex (Fig. 3.A). The Ch4 showed positive FC with the extensive cerebral cortex, while we did not find negative connection (Fig. 3.B).

**Fig. 4:**
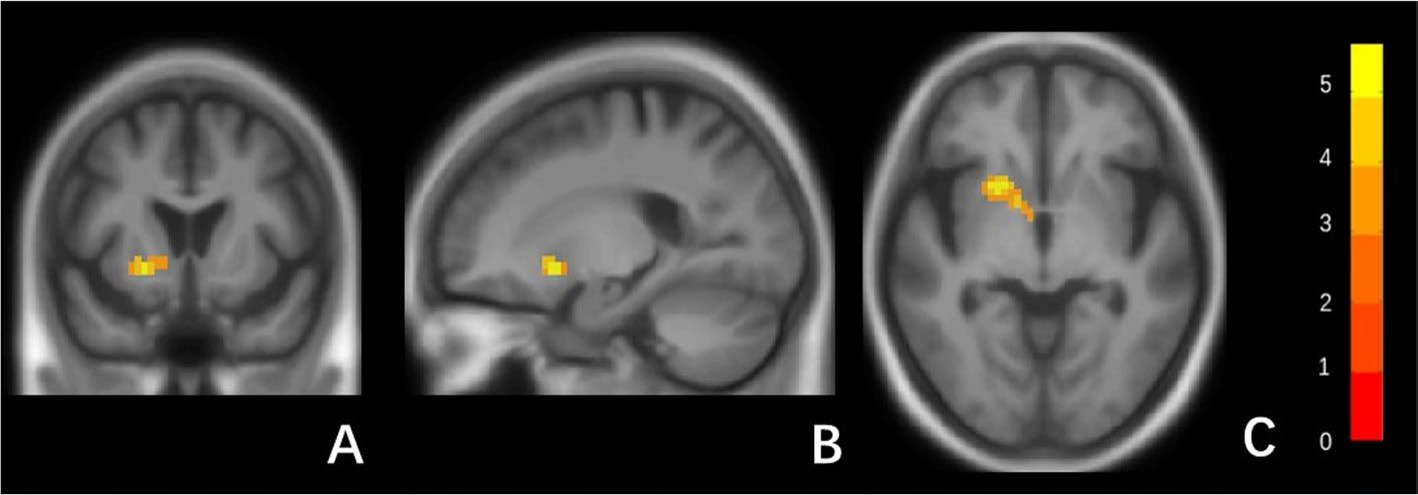
Statistical parametric map of the significant cerebral cortical clusters between two groups. Coronal (**A**), sagittal (**B**), and axial (**C**) view (MNI coordinate: x = − 21, y = 15, z = − 6) of significant different cluster between supernormal and typical normal participants (right of image is right of brain). The color bar in the right lower corner indicates the T-value

Figure 4 show the significant difference clusters in the FC for each seed between two groups. The superagers had stronger cerebral cortical FCs Ch1-3, compared to age, gender and education level matched typical older adults. The Ch1-3 had stronger connections with left putamen and insular cortex. Cerebral FC in Ch4 was not significantly different between two groups.

### Cognitive correlations of FC

**Fig. 5:**
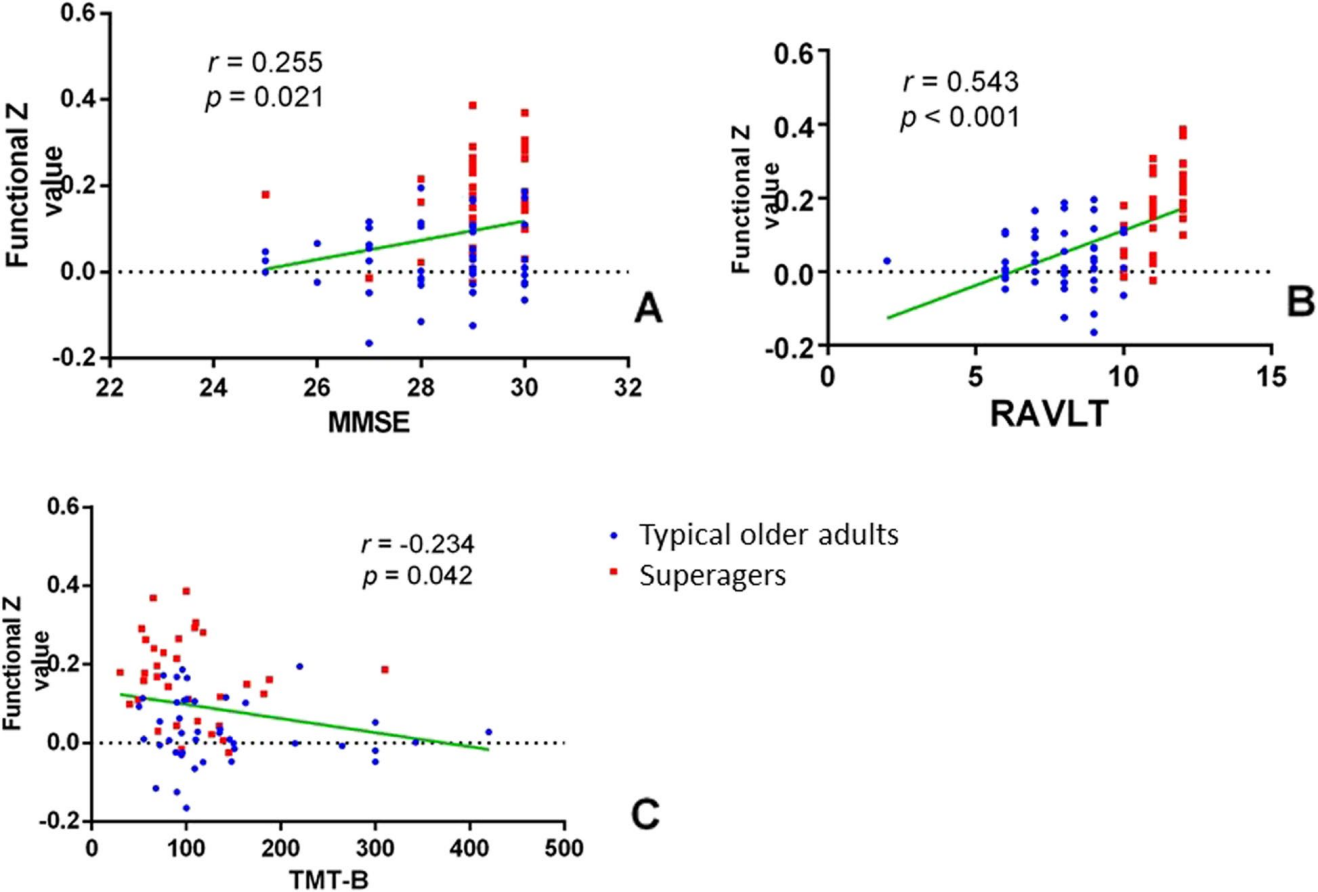
Scatter plots for the significant cognitive-functional connectivity (FC) correlations in all participants. Correlations between z-value of FC and clinical scores in Mini-mental State Examination (for global cognition), Delay recall of Rey Auditory-Verbal Learning Test (for memory), and Trail Making Test Part B (for executive function) in all participants, with age, gender, and education level as nuisance covariates.. The supernormals are showed in red, and the typical normal are showed in blue. Abbreviations: MMSE: Mini-mental State Examination; RAVLT: Rey Auditory-Verbal Learning Test; TMT-B: Trail Making Test Part

Cognitive correlates of the FC findings in all participants were investigated for the significant Ch1-3-cerebral FCs after controlling for age, gender and education (Table 2 and Fig. 5). The correlation for group with superagers and group with typical older adults is also described in Table 2. For Ch1-3, the strength of FC positively correlated with memory (Delay recall of RAVLT) in superagers. No significant correlation was found in typical older adults. However, when the group with superagers and group with typical older adults group combined together, cognitive performance in global cognition (MMSE), memory (Delay recall of RAVLT) and executive function (TMT-B) were found correlated with strength of FC in Ch 1-3.

**Table 2:** Correlation between cognition and FC

		Superagers	Typical older adults	All
MMSE Raw (total = 30)	***r/p***	0.255/0.146	0.059/0.689	**0.255/0.021**
RAVLT Delay-Recall Raw (total = 15)	***r/p***	**0.605/< 0.001**	−0.035/0.812	**0.543/< 0.001**
BNT-30 Raw	***r/p***	−0.158/0.372	−0.087/0.556	0.026/0.815
Trail-Making Test Part A Raw (sec)	***r/p***	−0.173/0.335	−0.102/0.497	− 0.123/0.273
Trail-Making Test Part B Raw (sec)	***r/p***	−0.089/0.616	−0.104/0.506	**− 0.234/0.042**
WAIS-III Block Design	***r/p***	0.089/0.618	0.071/0.637	0.187/0.095

*Abbreviations*: *MMSE* Mini-Mental State Examination, *RAVLT* Rey Auditory-Verbal Learning Test, *BNT* Boston Naming Test, *WAIS* Wechsler Adult Intelligence Scale. Bold means *p* < 0.05

## Discussion

The cerebral structural and functional change associated with aging play an important role in memory impairments in elder people. It’s still unclear the exact mechanism of cholinergic system in memory reservation in superagers. In this study, we found that the superagers had stronger cerebral cortical FCs of Ch1-3 with left putamen and insular cortex, and the strength of FC positively correlated with global cognition, memory and executive function.

How to define the cut-off of age for superagers is not consistent in previous studies. In this study, the definition of “superagers” is based on individual memory performance compared to age matched peers. According to the characteristics of age distribution in our sample, we select the participants with age over 60 years, which is adopted in seven studies [1, 3, 5–7, 11, 30]. Another problem is that the definition of “typical older adults” is not consistent either. In this study, we require the typical older adults to score within 1 SD of the average range for their age and education on each of these neuropsychological tests according to published normative values, which is used by Harrison et al. [9]. In addition, we also exclude the participants with left handed, in order to avoid the brain lateralization for cognitive processing.

Previous studies showed the atrophy of basal forebrain and its connected cortex, including hippocampus and precuneus, in patients with mild cognitive impairment (MCI) and AD [21, 31]. Longitudinal study also found the Ch4 atrophy in amnestic MCI predict the longitudinal memory decline [32]. Subjective cognitive decline (SCD) is considered as the incipient AD. Scheef et al. found atrophy of posterior basal forebrain in SCD, suggesting that the early involvement of cholinergic basal forebrain is already emerging in the preclinical stage of AD [33]. These previous findings reflect that the degeneration of cholinergic basal forebrain begins from the preclinical stage of AD. Our study may extend the observation towards aged people with normal cognition by showing the superagers had stronger basal forebrain FC than typical older adults. These findings indicate that successful aging is related with resistance and resilience to AD-related changes in cholinergic system, which may provide a new insight for cognitive preservation in older age.

In contrast to earlier findings, however, we did not find morphometric difference of basal forebrain between groups in this study. This inconsistency may be due to the participants in this study are people who do not have subjective or objective cognitive decline. Another possible explanation is that the altered FC could be due to the dysfunction of neurotransmitter or network connection, instead of being secondary to the atrophy.

In this study, the Ch1-3 had stronger connections with left putamen and insular cortex. A recent study found the association between attention/executive function and dopaminergic activity of putamen [34]. Our study suggested that the cholinergic activity of putamen also correlated with cognition. The individual-difference in cognitive function which correlated with the FC change of Ch1-3 circuits also agreed with the anticholinergic drugs induced cognitive decline in patients with Parkinson’s disease. In Parkinson’s disease, the anticholinergic drugs will impair immediate recall of information by influencing the immediate registration of information [35]. Furthermore, previous research found that cholinergic innervation is associated with enhancing perceptual discrimination of sensory input, which might contribute to the encoding of novel information [36]. One unanticipated finding was that the FC between Ch1-3 and insular cortex were correlated with the cognition function. The insula cortex is traditionally considered as the core area of sensorimotor processing, socio-emotional processing and central-olfactogustatory. However, with the development of functional neuroimaging methods, there is considerable evidence suggesting that the insular cortex is also involved in attention and salience processing [37]. However, the involvement of insular cortex must be interpreted with caution because the significant cluster was a very small region, only a few voxels extending into the white matter near insular cortex. Another interesting find is the lateralization of the results. This is may be due to the small sample size in this study. In fact, when the threshold of uncorrected *p <* 0.005 was chose to explore the brain regions, bilateral insular cortex would have stronger FC in superagers.

There are some limitations to this study. First, it’s an exploratory study with small sample size. Though the operational classification of “superaging” is inconsistent between studies, we were unable to compare the results with different neuropsychological criteria of “superaging”. Second, this was a retrospective cross-sectional study with a relatively small sample, and all the participants were selected from a memory clinic and were eligible for rs-fMRI, which will induce sampling bias. In addition, both of the groups had larger percentage of female participants, and most of the participants had college degree, possibly making the study less generalizable. Prospective studies with larger sample are needed to investigate the longitudinal effects of volume and function of basal forebrain in superagers. Third, we did not include the genetic influences, such as the Apolipoprotein (ApoE)-ε4 allele, which may yield the resistance to the aging related anatomic and functional change in brain.

## Conclusions

In conclusion, we found that the stronger FC of basal forebrain correlated with specific cognitive difference in global cognition and domains of memory and executive function in superagers. These findings provide further evidence that resistant of cholinergic basal forebrain degeneration is an important mechanism underlying successful aging.

## Data Availability

The dataset is available at 10.17632/bvkns2xxn2.1

## Abbreviations

AD: Alzheimer’s disease
ApoE: Apolipoprotein
BNT: Boston Naming Test
CSF: Cerebrospinal fluid tissue
DPARSF: Data Processing Assistant for Resting-State fMRI
FC: Functional connectivity
FD: Frame-wise displacement
FDR: False discovery rate ()
FOV: Field of view
FSPGR: Fast spoiled gradient-echo sequences
FWHM: Full width at half maximum
GM: Gray matter
MCI: Mild cognitive impairment
MMSE: Mini-Mental State Examination
MNI: Montreal Neurological Institute
MOFC: Medial orbitofrontal cortex
MPFC: Medial prefrontal cortex
NbM: Nucleus basalis of Meynert
RAVLT: Rey Auditory-Verbal Learning Test
REST: Resting-State fMRI Data Analysis Toolkit
ROI: Region-of-interest
SCD: Subjective cognitive decline
SD: Standard deviation
TE: Echo time
TMT: Trail Making Test
TR: Repetition time
VBM: Voxel-based morphometry
WAIS: Wechsler Adult Intelligence Scale
WM: White matter
